# Traditional Uses and Pharmacologically Active Constituents of *Dendrobium* Plants for Dermatological Disorders: A Review

**DOI:** 10.1007/s13659-021-00305-0

**Published:** 2021-04-20

**Authors:** Yue-Hu Wang

**Affiliations:** Key Laboratory of Economic Plants and Biotechnology, The Yunnan Key Laboratory for Wild Plant Resources, and Bio-Innovation Center of DR PLANT, Kunming Institute of Botany, Chinese Academy of Sciences, Kunming, Yunnan 650201 People’s Republic of China

**Keywords:** Orchidaceae, *Dendrobium*, Traditional uses, Dermatological disorders, Anti-inflammatory

## Abstract

*Dendrobium* Sw. is one of the largest genera in the orchidaceous family and includes 900–2000 species. Among them, more than 80 *Dendrobium* species have been reported in China. However, there are only six *Dendrobium* species, namely, *D. bigibbum* var. *superbum* (syn. *D. phalaenopsis*), *D. chrysanthum*, *D. fimbriatum*, *D. loddigesii*, *D. nobile*, and *D. officinale* (syn. *D. candidum*), listed in the New Inventory of Existing Cosmetic Ingredients in China Launched. Artificial planting of *Dendrobium* species has been a great success in China. To better utilize *Dendrobium* resources for medicinal and cosmetic purposes, we summarize their traditional uses and pharmacologically active compounds for treating dermatological disorders in this review. “Orchidaceae”, “*Dendrobium*”, “traditional use”, “ethnobotany”, “dermatological disorder”, and “skin disease” were used as search terms to screen the literature. Cited references were collected between 1970 and 2020 from the Web of Science, China National Knowledge Internet (CNKI), SciFinder, Google Scholar, and Chinese books. From the search, it was found that there are 22 *Dendrobium* species with traditional uses in dermatological disorders, and 131 compounds from *Dendrobium* plants have been reported to possess anti-inflammatory, antimicrobial, antioxidant, antiaging, anti-psoriasis, and tyrosinase-inhibitory activities, implying that *Dendrobium* plants are important resources for the discovery of active compounds and the development of new drugs and cosmetics. *D. crepidatum*, *D. denneanum*, *D. loddigesii*, *D. nobile*, and *D. officinale* have been extensively studied. More research on other *Dendrobium* species is needed. The major active compounds found in *Dendrobium* species are phenanthrenes, alkaloids, flavonoids, phenylpropanoids, and lignans. Several compounds, such as loddigesiinol A, (*S*)-5-methoxy-2,4,7,9-tetrahydroxy-9,10-dihydrophenanthrene, (*S*)-4-methoxy-2,5,7,9-tetrahydroxy-9,10-dihydrophenanthrene, 2,5-dihydroxy-4-methoxy-phenanthrene 2-*O*-*β*-d-glucopyranoside, (9*R*)-1,2,5,9-tetrahydroxy-9,10-dihydrophenanthrene 5-*O*-*β*-d-glucopyranoside, (+)-homocrepidine A, and vicenin 2, have significant anti-inflammatory activities and inhibit nitric oxide (NO) production with IC_50_ values less than 5 μM, and these compounds are worthy of further study.

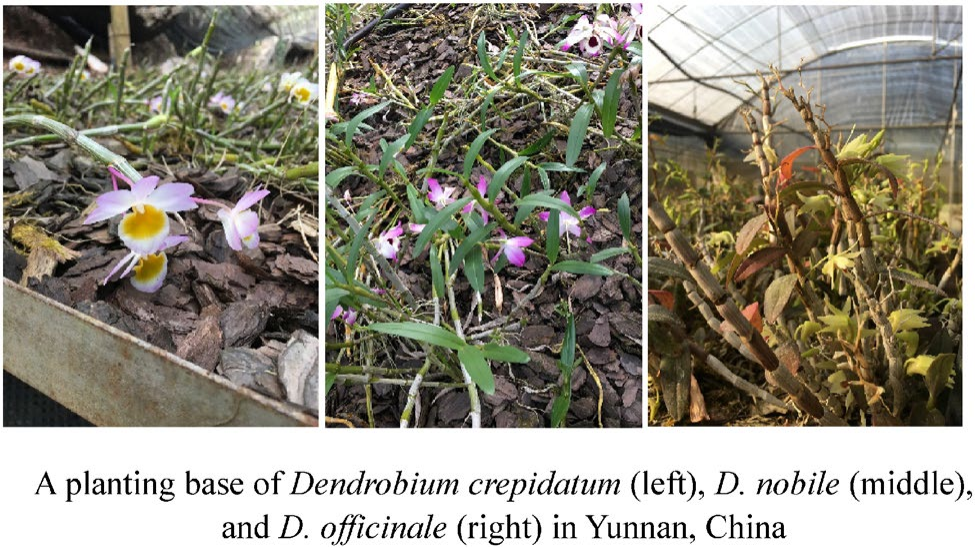

## Introduction

Even though human skin is not the largest organ by weight or functional surface area [1], it is the main interface of the human body with the external environment. The skin meets the environment directly and thus is vulnerable to various types of damage. For this function, the skin possesses remarkable barrier qualities that protect humans from external pathogens and prevent the uncontrolled loss of water from the body. Although mortality rates for skin diseases are generally relatively low, they are often persistent, difficult to treat, can significantly impact quality of life and have a major psychological impact [2].

*Dendrobium* Sw. is one of the most important genera of Orchidaceae. The genus is one of the largest members of the orchidaceous family and includes 900–2000 species [3]. *Dendrobium* plants are mainly distributed in the tropics and subtropics in southern Asia, Oceanica, and elsewhere [4]. According to *Plants of the World Online*, there are 1,556 accepted *Dendrobium* species at present [5]. Among them, more than 80 *Dendrobium* species have been reported in China [4]. However, there are only six *Dendrobium* species, namely, *D. bigibbum* var. *superbum* Rchb.f. (syn. *D. phalaenopsis* Fitzg.), *D. chrysanthum* Wall. ex Lindl., *D. fimbriatum* Hook., *D. loddigesii* Rolfe, *D. nobile* Lindl, and *D. officinale* Kimura et Migo (syn. *D. candidum* Wall. ex Lindl.), listed in the New Inventory of Existing Cosmetic Ingredients in China Launched (IECIC 2015, Final Version) [6].

Artificial planting of *Dendrobium* species has been a great success in China. The total mass of annual cultivated *Dendrobium* plants in China now exceeds 19 million kg [7]. To better utilize *Dendrobium* resources for medicinal and cosmetic purposes, we summarize their traditional uses and pharmacologically active constituents for treating dermatological disorders. “Orchidaceae”, “*Dendrobium*”, “traditional use”, “ethnobotany”, “dermatological disorder”, and “skin disease” were used as search terms to screen the literature. Cited references were collected between 1970 and 2020 from the Web of Science, China National Knowledge Internet (CNKI), SciFinder, Google Scholar, and Chinese books. For pharmacological activities, only extracts or compounds with IC_50_, EC_50_, or MIC values less than 100 μg/mL were cited.

## Traditional Uses of *Dendrobium* Species for Treating Dermatological Disorders

Traditional uses of 22 *Dendrobium* species for treating dermatological disorders by local people in Australia, Bangladesh, China, India, Indonesia, Liberia, Malaysia, and Nepal are found in the literature (Table 1).

**Table 1:** Traditional uses of *Dendrobium* plants for treating dermatological disorders in different countries

Latin name	Country	Local name	Part used	Traditional use	References
*Dendrobium affine* Steudel	Australia	Marndaja, tjalamarinj	Pseudobulbs	The sap from the pseudobulbs is directly squeezed onto sores to relieve itchy skin	[85]
	Australia	–	Stems, bulbs	Fluid from the stem or bulb is used on skin to treat itching, cuts, sores and minor burns	[86]
*Dendrobium alpestre* Royle	India	Jiwanti	Bulbs	For treating pimples, boils and other skin eruptions	[87]
*Dendrobium amoenum* Wall. ex Lindl	India	–	Leaves	Leaf paste is used to treat skin diseases	[88]
	India	Mitha alu	Leaves	Leaves of *D. amoenum* pounded with *Hedychium wardii* C.E.C. Fisch. rhizomes are made into a paste, which is used to treat wounds and various skin diseases	[89]
	Nepal	Thuur	Pseudobulbs	A fresh paste is applied topically on burnt skin	[90]
*Dendrobium aphyllum* (Roxb.) C.E.C. Fisch	Bangladesh	–	Leaves	A paste or juice of the leaves is used to treat wounds	[91]
	China	Dou chun shi hu	Whole plants, stems	Whole plants are used to treat burns and scalds. Fresh stems are externally used to tread burns and scalds	[92, 93]
	India	–	Leaves	Fresh leaf juice is used to treat skin infections	[94]
*Dendrobium denneanum* Kerr [syn. *Dendrobium aurantiacum* var. *denneanum* (Kerr) Z.H.Tsi]	China	Die qiao shi hu	Stems, leaves	Stems are used to treat impetigo. Dry leaves are externally used to treat impetigo	[92, 95]
*Dendrobium canaliculatum* R. Br	Australia	Marndaja	Pseudobulbs	Pseudobulbs are squeezed, and the sap is applied directly to sores to help heal them	[85]
*Dendrobium chrysanthum* Wall. ex Lindl	India	Nauawimu	Stems	Stem juice is applied on wounds and sores	[96]
*Dendrobium crumenatum* Sw	Malaysia	Daun sepuleh tulang	Leaves	A poultice made from leaves is used to treat boils and pimples	[7]
*Dendrobium densiflorum* Lindl	Nepal	Sungava	Pseudobulbs	Fresh pulp is applied to boils and pimples	[90]
*Dendrobium discolor* Lindl	Australia	–	Stems	A poultice is prepared from young canes to draw a boil. A liniment made from mature canes is used to treat ringworm	[97]
*Dendrobium fimbriatum* Hook	India	–	Leaves	A paste of fresh leaves is used to treat boils and pimples	[94]
	India^a^	Mokya tu	Leaves	Approximately 10 g of leaves are ground, made into a paste and applied twice a day for 10 days to heal cuts and wounds	[98]
*Dendrobium hancockii* Rolfe	China	Xi ye shi hu	Stems	To treat ulcers	[99]
*Dendrobium herbaceum* Lindl	India	Agai	Roots	Fresh roots are burnt, and 10 g of the resultant ash is mixed with 10 ml mustard oil and applied on affected skin 2 to 3 times daily for several days until symptoms disappear	[100]
*Dendrobium macraei* Lindl. [syn. *Flickingeria macraei* (Lindl.) Seidenf.]	India	Sakar	Roots	One spoonful of a root paste along with 1 g of a seed powder of black pepper is administered orally on an empty stomach twice a day for 21 days to cure diseases, including skin allergies, and is applied on the affected part of skin to cure eczema	[101]
*Dendrobium macrostachyum* Lindl	India	Yanaimiratti	Aerial parts	Aerial parts are used for skin allergies	[102]
*Dendrobium monticola* P.F. Hunt & Summerh	Nepal	Jiwanti	Bulbs	For treating pimples, boils, and other skin eruptions	[103]
*Dendrobium nobile* Lindl	Bangladesh	–	Leaves, seeds	A leaf extract is made and is very effective for treating freshly cut wounds. A seed powder is used to cure cuts and wounds	[104]
	India	–	Pseudobulbs	A pseudobulb extract is used to soothe burns	[105]
	India	–	Roots, seeds	Powdery seeds and root powder are used to heal wounds	[106]
	India	–	Seeds	Powdery seeds are applied to fresh wounds for quick healing	[107]
	India	–	Whole plants	Whole plant parts are used in the treatment of cuts and wounds	[108]
*Dendrobium ovatum* (L.) Kraenzl	India	Unnesh chedi	Pseudostems	The plant is an emollient	[109]
*Dendrobium planibulbe* Lindl	Malaysia	Miga	–	A poultice is made by pounding the plant to treat dermatological lesions affecting the back of the neck	[7]
*Dendrobium polyanthum* Wall. ex Lindl. [syn. *Dendrobium primulinum* Lindl.]	China	Bao chun shi hu	Pseudobulbs	To treat burns and scalds, skin itching caused by a red rash, and eczema. Fresh pseudobulbs are ground with water to yield a juice, or fresh pseudobulbs are pounded and externally used to treat scalds. A decoction of dry pseudobulbs (15 g) is taken orally to treat skin itching caused by red rash	[110]
*Dendrobium purpureum* Roxb	Indonesia	–	Leaves, stems	To treat infected nails	[111]
*Dendrobium* sp.	Liberia	Gulubalama boblogie	Leaves	Crushed leaf extracts are applied on boils for fast relief	[112]

^a^In Ref. [98], the synonym *Dendrobium fimbriatum* Hook. var. *occulatum* Hook.f. of the plant was used

As shown in Table 1, the uses of *Dendrobium* plants include treatments of boils [*Dendrobium alpestre* Royle, *D. crumenatum* Sw., *D. densiflorum* Lindl., *D. discolor* Lindl., *D. fimbriatum*, *D. monticola* P.F. Hunt & Summerh., and *Dendrobium* sp. (local name: gulubalama boblogie)], cuts (*Dendrobium affine* Steudel, *D. fimbriatum*, and *D. nobile*), burns [*D. affine*, *D. amoenum* Wall. ex Lindl., *D. aphyllum* (Roxb.) C.E.C. Fisch., *D. nobile*, and *D. polyanthum* Wall. ex Lindl.], eczema (*D. macraei* Lindl. and *D. polyanthum*), impetigo (*D. denneanum* Kerr), infected nails (*Dendrobium purpureum* Roxb.), itchy skin (*Dendrobium affine* Steudel), pimples (*D. alpestre*, *D. crumenatum*, *D. densiflorum*, *D. fimbriatum*, and *D. monticola*), scalds (*D. aphyllum* and *D. polyanthum*), skin allergies (*D. macraei* and *D. macrostachyum* Lindl.), sores (*D. affine*, *D. canaliculatum* R. Br., and *D. chrysanthum*), ringworm (*D. discolor*), ulcer (*D. hancockii* Rolfe), and wounds (*D. amoenum*, *D. aphyllum*, *D. chrysanthum*, *D. fimbriatum*, and *D. nobile*). The treatments of boils (seven species), burns (five species), pimples (five species), and wounds (five species) are the most common uses (Table 1).

Plant parts used of *Dendrobium* species for treating dermatological disorders include aerial parts (one species), bulbs (three species), leaves (eight species), pseudobulbs (six species), pseudostems (one species), roots (three species), seeds (one species), stems (seven species), and whole plants (two species). Leaves, pseudobulbs, and stems are the most common parts used.

## Pharmacological Activities of Extracts, Preparations, and Chemical Constituents from *Dendrobium* Plants for Treating Dermatological Disorders

Some extracts, preparations, and chemical constituents from *Dendrobium* plants exhibit pharmacological activities related to dermatological disorders, such as anti-inflammatory, antimicrobial, antioxidant, antiaging, anti-psoriasis, hair growth promoting, skin-moisturizing, and tyrosinase-inhibitory activities. A part of pharmacological activities is related to the traditional uses of *Dendrobium* plants. For example, anti-inflammatory activities are associated with treatments of eczema, itchy skin, and skin allergies, while antimicrobial activities are associated with treatments of boils, impetigo, and pimples.

**Fig. 1:**
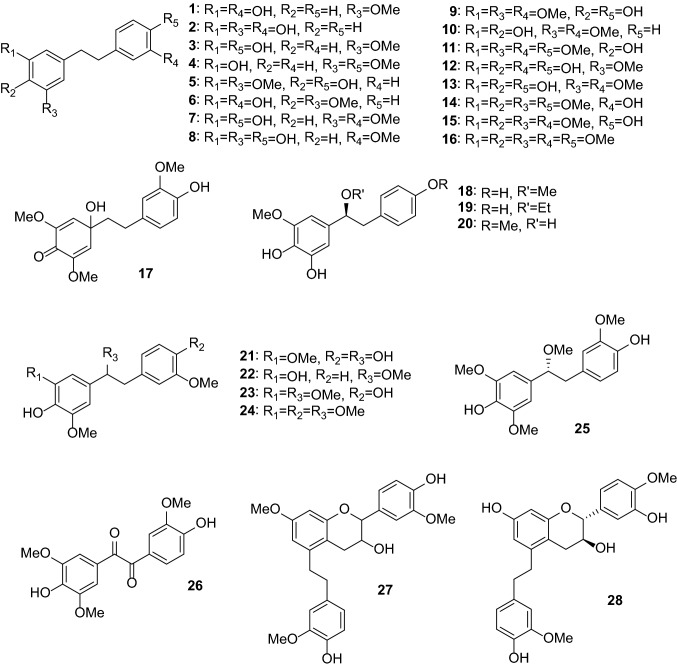
Chemical structures of pharmacologically active bibenzyls (**1**–**28**) from *Dendrobium* plants (I)

**Fig. 2:**
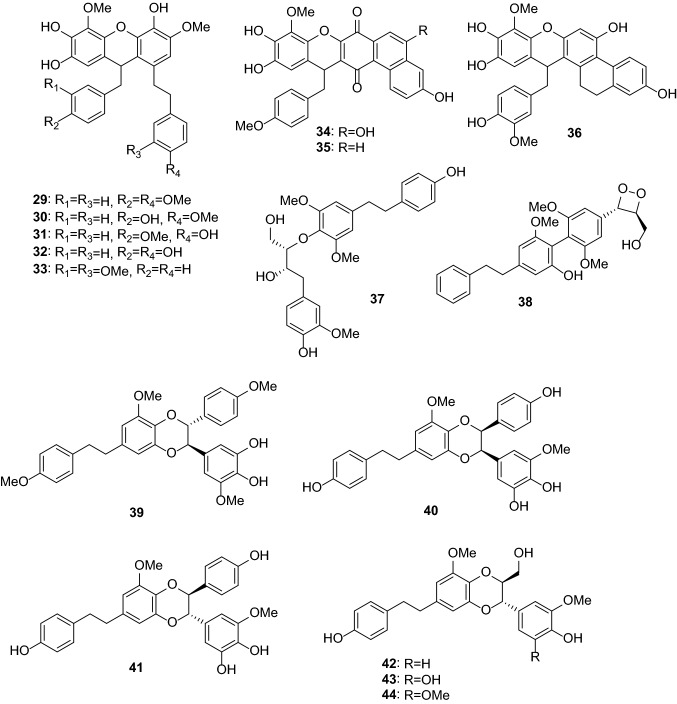
Chemical structures of pharmacologically active bibenzyls (**29**–**44**) from *Dendrobium* plants (II)

**Fig. 3:**
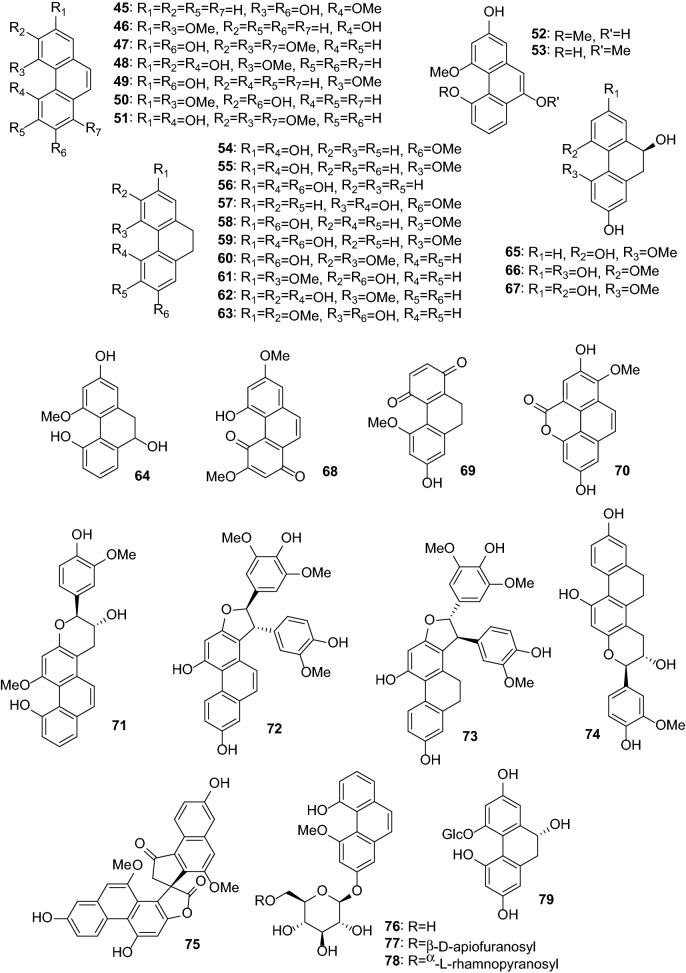
Chemical structures of pharmacologically active phenanthrenes (**45**–**79**) from *Dendrobium* plants

**Fig. 4:**
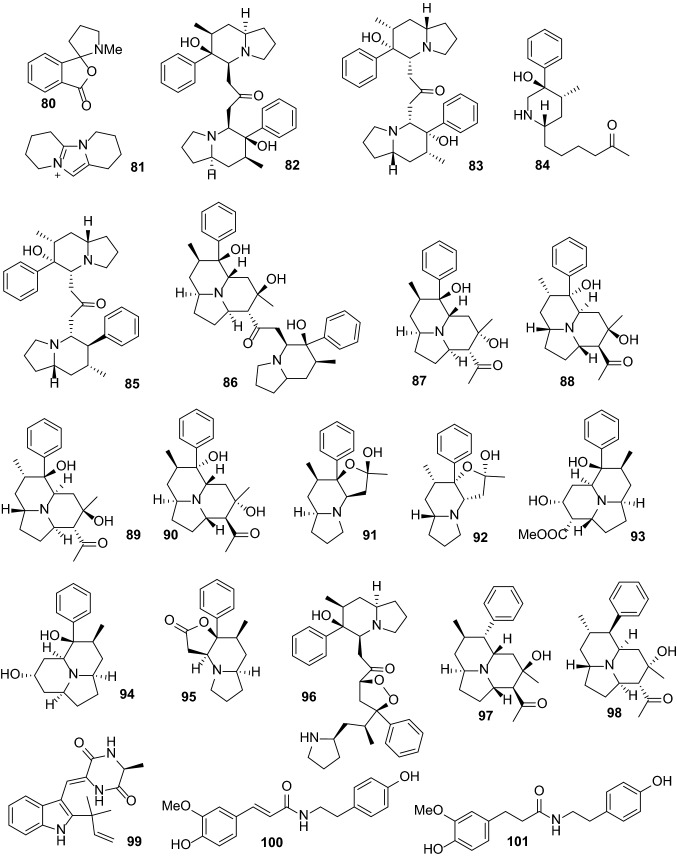
Chemical structures of pharmacologically active alkaloids (**80**–**101**) from *Dendrobium* plants

**Fig. 5:**
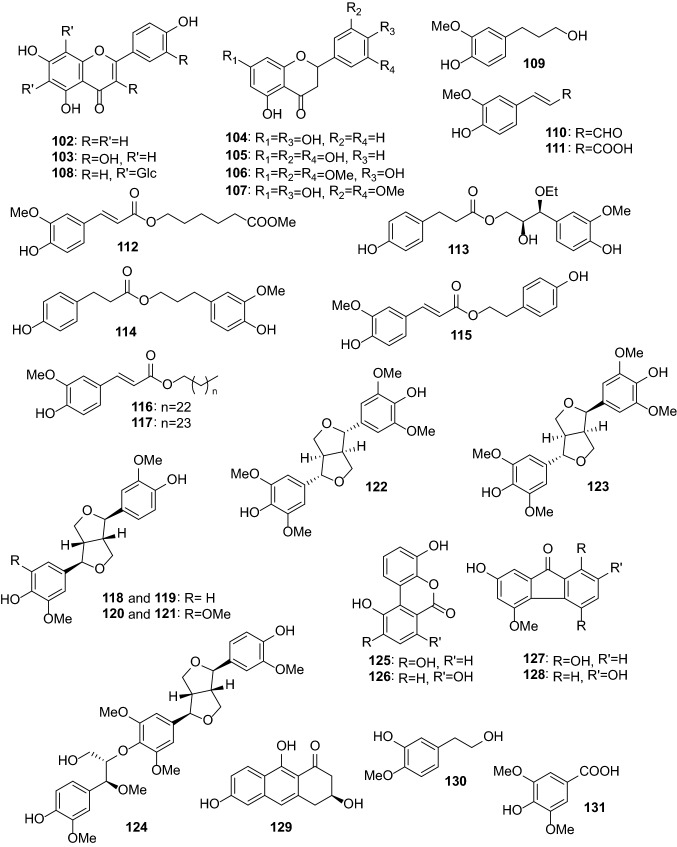
Chemical structures of pharmacologically active flavonoids (**102**–**108**), phenylpropanoids (**109**–**117**), lignans (**118**–**124**) and other active compounds (**125**–**131**) from *Dendrobium* plants

Anti-inflammatory and antioxidant activities are the most common activities of *Dendrobium* extracts and compounds. One hundred thirty-one compounds from *Dendrobium* plants have been reported to possess anti-inflammatory, antimicrobial, antioxidant, antiaging, anti-psoriasis, and tyrosinase-inhibitory activities (Table 2). These compounds include bibenzyls (**1**–**44**, Figs. 1 and 2), phenanthrenes (**45**–**79**, Fig. 3), alkaloids (**80**–**101**, Fig. 4), flavonoids (**102**–**108**), phenylpropanoids (**109**–**117**), lignans (**118**–**124**), and others (**125**–**131**, Fig. 5).

**Table 2:** Pharmacologically active compounds (**1**–**131**) from *Dendrobium* plants

No.	Name	Type	Source	Pharmacological activities^a^	References
**1**	Batatasin III	Bibenzyls	*D. loddigesii*	Antiaging (collagen production: EC_50_ 3.2 μg/mL), anti-inflammatory (NO: IC_50_ 21.9 μM), and antioxidant (DPPH: IC_50_ 50.1 μg/mL)	[20, 49]
**2**	3,3ʹ,5-Trihydroxybibenzyl	Bibenzyls	*D. loddigesii*	Anti-inflammatory (NO: IC_50_ 13.1 μM) and antioxidant (DPPH: 85.8 μM)	[20]
				Tyrosinase-inhibitory (IC_50_ 37.9 μg/mL)	[49]
**3**	3,4ʹ-Dihydroxy-5-methoxybibenzyl	Bibenzyls	*D. officinale*	Antioxidant (ABTS: 5.3 μM)	[66]
**4**	3-Hydroxy-4ʹ,5-dimethoxybibenzyl	Bibenzyls	*D. heterocarpum*	Anti-inflammatory	[15]
**5**	4,4ʹ-Dihydroxy-3,5-dimethoxybibenzyl	Bibenzyls	*D. loddigesii*	Anti-inflammatory (NO: IC_50_ 49.3 μM) and antioxidant (DPPH: IC_50_ 94.5 μM)	[20]
**6**	3,3ʹ-Dihydroxy-4,5-dimethoxybibenzyl	Bibenzyls	*D. williamsonii*	Antioxidant (DPPH: IC_50_ 19.5 μM)	[74]
**7**	Gigantol	Bibenzyls	*D. draconis*	Antioxidant (DPPH: IC_50_ 17.7 μM)	[47]
			*D. heterocarpum*	Anti-inflammatory	[15]
			*D. loddigesii*	Antioxidant	[49]
			*D. nobile*	Anti-inflammatory (NO: IC_50_ 32.9 μM) and antioxidant (DPPH: IC_50_ 56.4 μM)	[23]
			*Dendrobium* species	Antibacterial (*Staphylococcus aureus*: MIC 82.2 μg/mL)	[32, 33]
**8**	Tristin	Bibenzyls	*D. loddigesii*	Antioxidant	[49]
			*D. officinale*	Antioxidant (ABTS: IC_50_ 9.0 μΜ; DPPH: IC_50_ 34.5 μM)	[66]
**9**	Moscatilin	Bibenzyls	*D. loddigesii*	Antioxidant	[49]
			*D. nobile*	Anti-inflammatory (NO: IC_50_ 27.6 and 36.8 μM) and antioxidant (DPPH: IC_50_ 14.5 μM)	[22, 23]
			*D. secundum*	Antioxidant (DPPH: IC_50_ 5.1 μM)	[70]
			*D. williamsonii*	Antioxidant (DPPH: IC_50_ 8.5 μM)	[74]
**10**	Dendrobin A	Bibenzyls	*D. nobile*	Antioxidant (DPPH: IC_50_ 40.3 μM)	[23]
**11**	Chrysotoxine	Bibenzyls	*D. nobile*	Antioxidant (DPPH: IC_50_ 14.0 μM)	[23]
**12**	Dendrocandin E	Bibenzyls	*D. officinale*	Antioxidant (DPPH: IC_50_ 15.6 μM)	[61]
**13**	4,5,4ʹ-Trihydroxy-3,3ʹ-dimethoxybibenzyl	Bibenzyls	*D. loddigesii*	Antioxidant	[49]
			*D. secundum*	Antioxidant (DPPH: IC_50_ 15.9 μM)	[70]
**14**	Erianin	Bibenzyls	*D. chrysotoxum*	Antibacterial (srtA: IC_50_ 20.9 μg/mL)	[34, 35]
				Anti-psoriasis	[75]
**15**	Crepidatin	Bibenzyls	*D. loddigesii*	Antioxidant	[49]
			*D. nobile*	Antioxidant (DPPH: IC_50_ 21.8 μM)	[23]
**16**	Chrysotobibenzyl	Bibenzyls	*D. nobile*	Anti-inflammatory (NO: IC_50_ 48.2 μM)	[23]
**17**	Aphyllone B	Bibenzyls	*D. aphyllum*	Antioxidant	[42]
**18**	Dendrocandin C	Bibenzyls	*D. officinale*	Antioxidant (DPPH: IC_50_ 34.2 μM)	[61]
**19**	Dendrocandin D	Bibenzyls	*D. officinale*	Antioxidant (DPPH: IC_50_ 34.5 μM)	[61]
**20**	(*S*)-3,4,*α*-trihydroxy-5,4ʹ-dimethoxybibenzyl	Bibenzyls	*D. officinale*	Antioxidant (DPPH: IC_50_ 32.3 μM)	[64]
**21**	Nobilin D	Bibenzyls	*D. nobile*	Anti-inflammatory (NO: IC_50_ 15.3 μM) and antioxidant (DPPH: IC_50_ 19.9 μM)	[23]
**22**	Nobilin A	Bibenzyls	*D. nobile*	Antioxidant (DPPH: IC_50_ 87.1 μM)	[56]
**23**	Nobilin B	Bibenzyls	*D. nobile*	Antioxidant (DPPH: IC_50_ 32.2 μM)	[56]
**24**	Nobilin C	Bibenzyls	*D. nobile*	Antioxidant (DPPH: IC_50_ 47.4 μg/mL)	[56]
**25**	Loddigesiinol C	Bibenzyls	*D. loddigesii*	Antioxidant (DPPH: IC_50_ 23.7 μM)	[19]
**26**	Loddigesiinol D	Bibenzyls	*D. loddigesii*	Anti-inflammatory (NO: IC_50_ 69.7 μM)	[19]
**27**	Crepidatuol B	Bibenzyls	*D. loddigesii*	Antioxidant	[50]
**28**	Trigonopol B	Bibenzyls	*D. loddigesii*	Anti-inflammatory (NO: IC_50_ 26.3 μM) and antioxidant (DPPH: IC_50_ 60.1 μM)	[20]
**29**	Dendrocandin F	Bibenzyls	*D. officinale*	Antioxidant (DPPH: IC_50_ 55.8 μM)	[62]
**30**	Dendrocandin G	Bibenzyls	*D. officinale*	Antioxidant (DPPH: IC_50_ 32.4 μM)	[62]
**31**	Dendrocandin J	Bibenzyls	*D. officinale*	Aantioxidant (DPPH: IC_50_ 36.8 μM)	[63]
**32**	Dendrocandin K	Bibenzyls	*D. officinale*	Antioxidant (DPPH: IC_50_ 70.2 μM)	[63]
**33**	Nobilin E	Bibenzyls	*D. nobile*	Anti-inflammatory (NO: IC_50_ 19.2 μM) and antioxidant (DPPH: IC_50_ 21.0 μM)	[23]
**34**	Dendrocandin H	Bibenzyls	*D. officinale*	Antioxidant (DPPH: IC_50_ 32.4 μM)	[62]
**35**	Dendrocandin L	Bibenzyls	*D. officinale*	Antioxidant (DPPH: IC_50_ 45.0 μM)	[63]
**36**	(−)-Dendroparishiol	Bibenzyls	*D. parishii*	Anti-inflammatory	[28]
**37**	Dendrocandin M	Bibenzyls	*D. officinale*	Antioxidant (DPPH: IC_50_ 60.5 μM)	[63]
**38**	6ʺ-De-*O*-methyldendrofindlaphenol A	Bibenzyls	*D. findlayanum*	Anti-inflammatory (NO: IC_50_ 21.4 μM)	[14]
**39**	Dendrocandin I	Bibenzyls	*D. heterocarpum*	Anti-inflammatory	[15]
			*D. officinale*	Antioxidant (DPPH: IC_50_ 21.3 μM)	[62]
**40**	Dendrocandin P	Bibenzyls	*D. officinale*	Antioxidant (DPPH: IC_50_ 22.3 μM)	[63]
**41**	Dendrocandin Q	Bibenzyls	*D. officinale*	Antioxidant (DPPH: IC_50_ 30.3 μM)	[63]
**42**	Dendrocandin N	Bibenzyls	*D. officinale*	Antioxidant (DPPH: IC_50_ 87.6 μM)	[63]
**43**	Dendrocandin O	Bibenzyls	*D. officinale*	Antioxidant (DPPH: IC_50_ 50.4 μM)	[63]
**44**	Dendrocandin U	Bibenzyls	*D. officinale*	Antioxidant (ABTS: IC_50_ 10.0 μM)	[66]
**45**	Moscatin (plicatol B)	Phenanthrenes	*D. denneanum*	Anti-inflammatory (NO: IC_50_ 6.3 μM)	[13]
			*D. loddigesii*	Anti-inflammatory (NO: IC_50_ 6.4 μM) and antioxidant (DPPH: IC_50_ 59.8 μM)	[19, 50]
**46**	5-Hydroxy-2,4-dimethoxyphenanthrene	Phenanthrenes	*D. loddigesii*	Anti-inflammatory (NO: IC_50_ 5.3 μM)	[19]
**47**	Confusarin	Phenanthrenes	*D. nobile*	Antioxidant (DPPH: IC_50_ 12.9 μM)	[57]
**48**	Fimbriol B	Phenanthrenes	*D. nobile*	Anti-inflammatory (NO: IC_50_ 28.9 μM)	[22]
**49**	Flavanthrinin	Phenanthrenes	*D. nobile*	Antioxidant (DPPH: IC_50_ 35.7 μM)	[57]
**50**	5,7-Dimethoxyphenanthrene-2,6-diol	Phenanthrenes	*D. nobile*	Anti-inflammatory (NO: IC_50_ 37.7 μM)	[22]
				Antioxidant (DPPH: IC_50_ 29.7 μM)	[57]
**51**	3,4,8-Trimethoxyphenanthrene-2,5-diol	Phenanthrenes	*D. nobile*	Anti-inflammatory (NO: IC_50_ 20.4 μM)	[22]
**52**	Loddigesiinol A	Phenanthrenes	*D. loddigesii*	Anti-inflammatory (NO: IC_50_ 2.6 μM) and antioxidant (DPPH: IC_50_ 26.1 μM)	[19]
**53**	2,5-Dihydroxy-4,9-dimethoxyphenanthrene	Phenanthrenes	*D. nobile*	Antioxidant (DPPH: IC_50_ 34.8 μM)	[57]
**54**	Lusianthridin	Phenanthrenes	*D. loddigesii*	Anti-inflammatory (NO: IC_50_ 4.6 μM) and antioxidant (IC_50_ 62.2 μM)	[19]
			*D. nobile*	Anti-inflammatory (NO: IC_50_ 9.6 μM)	[22]
**55**	Hircinol	Phenanthrenes	*D. draconis*	Antioxidant (DPPH: IC_50_ 22.3 μM)	[47]
			*D. loddigesii*	Anti-inflammatory (NO: IC_50_ 29.2 μM)	[19]
			*D. nobile*	Anti-inflammatory (NO: IC_50_ 26.4 μM)	[22]
**56**	9,10-Dihydrophenanthrene-2,4,7-triol	Phenanthrenes	*D. denneanum*	Anti-inflammatory (NO: IC_50_ 32.7 μM)	[13]
			*D. loddigesii*	Anti-inflammatory (NO: IC_50_ 8.6 μM) and antioxidant (DPPH: IC_50_ 14.1 μM)	[20, 50]
57	2-Methoxy-9,10-dihydrophenanthrene-4,5-diol	Phenanthrenes	*D. denneanum*	Anti-inflammatory (NO: IC_50_ 7.6 μM)	[13]
**58**	Coelonin	Phenanthrenes	*D. nobile*	Anti-inflammatory (NO: IC_50_ 10.2 μM)	[22]
**59**	7-Methoxy-9,10-dihydrophenanthrene-2,4,5-triol	Phenanthrenes	*D. draconis*	Antioxidant (DPPH: IC_50_ 10.2 μM)	[47]
**60**	Erianthridin	Phenanthrenes	*D. nobile*	Anti-inflammatory (NO: IC_50_ 19.5 μM)	[22]
**61**	Flavanthridin	Phenanthrenes	*D. nobile*	Anti-inflammatory (NO: IC_50_ 34.1 μM)	[22]
**62**	Epheneranthol C	Phenanthrenes	*D. nobile*	Anti-inflammatory (NO: IC_50_ 17.6 μM)	[22]
**63**	Ephemeranthol A	Phenanthrenes	*D. nobile*	Anti-inflammatory (NO: IC_50_ 12.0 μM)	[22]
**64**	Rotundatin (plicatol C)	Phenanthrenes	*D. loddigesii*	Anti-inflammatory (NO: IC_50_ 29.1 μM)	[19]
**65**	(*S*)-4-Methoxy-9,10-dihydrophenanthrene-2,5,9-triol	Phenanthrenes	*D. denneanum*	Anti-inflammatory (NO: IC_50_ 27.4 μM)	[13]
**66**	(*S*)-5-Methoxy-2,4,7,9-tetrahydroxy-9,10-dihydrophenanthrene	Phenanthrenes	*D. denneanum*	Anti-inflammatory (NO: IC_50_ 3.1 μM)	[13]
**67**	(*S*)-4-Methoxy-2,5,7,9-tetrahydroxy-9,10-dihydrophenanthrene	Phenanthrenes	*D. denneanum*	Anti-inflammatory (NO: IC_50_ 4.2 μM)	[13]
**68**	Denbinobin	Phenanthrenes	*D. moniliforme*	Anti-inflammatory	[21]
**69**	5-Methoxy-7-hydroxy-9,10-dihydro-1,4-phenanthrenequinone	Phenanthrenes	*D. draconis*	Antioxidant (DPPH: IC_50_ 72.6 μg/mL)	[47]
**70**	Fimbriatone	Phenanthrenes	*D. nobile*	Antioxidant (DPPH: IC_50_ 40.8 μg/mL)	[57]
**71**	Loddigesiinol B	Phenanthrenes	*D. loddigesii*	Anti-inflammatory (NO: IC_50_ 9.9 and 10.9 μM)	[19, 20]
**72**	Loddigesiinol I	phenanthrenes	*D. loddigesii*	Anti-inflammatory (NO: IC_50_ 7.5 μM)	[20]
**73**	Loddigesiinol J	Phenanthrenes	*D. loddigesii*	Anti-inflammatory (NO: IC_50_ 14.6 μM)	[20]
**74**	Chrysotoxol A	Phenanthrenes	*D. loddigesii*	Anti-inflammatory (NO: IC_50_ 10.9 μM) and antioxidant (DPPH: IC_50_ 23.2 μM)	[20]
**75**	Dendrochrysanene	Phenanthrenes	*D. chrysanthum*	Anti-inflammatory	[9]
**76**	2,5-Dihydroxy-4-methoxy-phenanthrene 2-*O*-*β*-d-glucopyranoside	Phenanthrenes	*D. denneanum*	Anti-inflammatory (NO: IC_50_ 4.6 μM)	[13]
**77**	2,5-Dihydroxy-4-methoxy-phenanthrene 2-*O*-*β*-d-apiofuranosyl-(1 → 6)-*β*-d-glucopyranoside	Phenanthrenes	*D. denneanum*	Anti-inflammatory (NO: IC_50_ 16.9 μM)	[13]
**78**	2,5-Dihydroxy-4-methoxy-phenanthrene 2-*O*-*α*-l-rhamnopyranosyl-(1 → 6)-*β*-d-glucopyranoside	Phenanthrenes	*D. denneanum*	Anti-inflammatory (NO: IC_50_ 41.5 μM)	[13]
**79**	(9*R*)-1,2,5,9-Tetrahydroxy-9,10-dihydrophenanthrene 5-*O*-*β*-d-glucopyranoside	Phenanthrenes	*D. denneanum*	Anti-inflammatory (NO: IC_50_ 0.7 μM)	[13]
**80**	Shihunine	Alkaloids	*D. loddigesii*	Anti-inflammatory (NO: IC_50_ 11.5 μg/mL)	[18]
**81**	Anosmine	Alkaloids	*D. nobile*	Anti-inflammatory (NO: IC_50_ 16.1 μg/mL)	[18]
**82**	(+)-Homocrepidine A	Alkaloids	*D. crepidatum*	Anti-inflammatory (NO: IC_50_ 3.6 μM)	[11]
**83**	(−)-Homocrepidine A	Alkaloids	*D. crepidatum*	Anti-inflammatory (NO: IC_50_ 22.8 μM)	[11]
**84**	Homocrepidine B	Alkaloids	*D. crepidatum*	Anti-inflammatory (NO: IC_50_ 27.6 μM)	[11]
**85**	(+)-Dendrocrepidamine A	Alkaloids	*D. crepidatum*	Anti-inflammatory (NO: IC_50_ 16.1 μM)	[12]
**86**	Dendrocrepidamine B	Alkaloids	*D. crepidatum*	Anti-inflammatory (NO: IC_50_ 14.3 μM)	[12]
**87**	(−)-Crepidine	Alkaloids	*D. crepidatum*	Anti-inflammatory (NO: IC_50_ 29.9 μM)	[12]
**88**	(+)-Crepidine	Alkaloids	*D. crepidatum*	Anti-inflammatory (NO: IC_50_ 81.9 μM)	[12]
**89**	(−)-Dendrocrepidine A	Alkaloids	*D. crepidatum*	Anti-inflammatory (NO: IC_50_ 18.5 μM)	[12]
**90**	(+)-Dendrocrepidine A	Alkaloids	*D. crepidatum*	Anti-inflammatory (NO: IC_50_ 30.2 μM)	[12]
**91**	(−)-Isocrepidamine	Alkaloids	*D. crepidatum*	Anti-inflammatory (NO: IC_50_ 16.3 μM)	[12]
**92**	(+)-Isocrepidamine	Alkaloids	*D. crepidatum*	Anti-inflammatory (NO: IC_50_ 73.0 μM)	[12]
**93**	Dendrocrepidine B	Alkaloids	*D. crepidatum*	Anti-inflammatory (NO: IC_50_ 51.8 μM)	[10]
**94**	Dendrocrepidine C	Alkaloids	*D. crepidatum*	Anti-inflammatory (NO: IC_50_ 29.7 μM)	[10]
**95**	Dendrocrepidine D	Alkaloids	*D. crepidatum*	Anti-inflammatory (NO: IC_50_ 40.1 μM)	[10]
**96**	Dendrocrepidine E	Alkaloids	*D. crepidatum*	Anti-inflammatory (NO: IC_50_ 35.2 μM)	[10]
**97**	(−)-Dendrocrepidine F	Alkaloids	*D. crepidatum*	Anti-inflammatory (NO: IC_50_ 13.3 μM)	[10]
**98**	(+)-Dendrocrepidine F	Alkaloids	*D. crepidatum*	Anti-inflammatory (NO: IC_50_ 42.7 μM)	[10]
**99**	Neoechinulin A	Alkaloids	*D. loddigesii*	Anti-inflammatory (NO: IC_50_ 50.0 μM)	[20]
**100**	Moupinamide	Alkaloids	*D. officinale*	Antioxidant (DPPH: IC_50_ 53.8 μM)	[65]
**101**	Dihydroferuloyltyramine	Alkaloids	*D. officinale*	Antioxidant (DPPH: IC_50_ 35.8 μg/mL)	[65]
**102**	Apigenin	Flavonoids	*D. williamsonii*	Antioxidant (DPPH: IC_50_ 19.3 μM)	[74]
**103**	Quercetin	Flavonoids	*D. tosaense*	Antioxidant	[54]
**104**	Naringenin	Flavonoids	*D. loddigesii*	Anti-inflammatory (NO: IC_50_ 26.9 μM)	[20]
**105**	3′,5,5′,7-Tetrahydroxyflavanone	Flavonoids	*D. officinale*	Antioxidant (DPPH: IC_50_ 29.8 μM)	[65]
**106**	5,4ʹ-Dihydroxy-7,3ʹ,5ʹ-trimethoxyflavanone	Flavonoids	*D. loddigesii*	Anti-inflammatory (NO: IC_50_ 24.9 μM) and antioxidant (DPPH: IC_50_ 78.9 μg/mL)	[20]
**107**	5,7,4ʹ-Trihydroxy-3ʹ,5ʹ-dimethoxyflavanone	Flavonoids	*D. loddigesii*	Anti-inflammatory (NO: IC_50_ 19.1 μM)	[20]
**108**	Vicenin 2 (vicenin II)	Flavonoids	*D. officinale*	Anti-inflammatory (TNF-*α*: IC_50_ 6.8 μM; NO: IC_50_ 3.9 μM)	[25, 26]
**109**	Dihydroconiferyl alcohol	Phenylpropanoids	*D. nobile*	Antioxidant (DPPH: IC_50_ 50.9 μM)	[56]
**110**	Coniferylaldehyde	Phenylpropanoids	*D. nobile*	Antioxidant (IC_50_ 22.8 μg/mL)	[56]
**111**	Ferulic acid	Phenylpropanoids	*D. officinale*	Antioxidant (DPPH: IC_50_ 64.9 μM)	[65]
			*D. secundum*	Antioxidant (DPPH: IC_50_ 37.5 μM)	[70]
**112**	6-Feruloyloxyhexanoic ester	Phenylpropanoids	*Dendrobium* cv. Sonia	Anti-inflammatory (NO: IC_50_ 29.6 μM)	[29]
**113**	*Threo*-7-*O*-ethyl-9-*O*-(4-hydroxyphenyl)propionyl-guaiacylglycerol	Phenylpropanoids	*D. loddigesii*	Antioxidant	[49]
**114**	Dihydroconiferyl dihydro-*p*-coumarate	Phenylpropanoids	*D. loddigesii*	Antioxidant	[49]
			*D. officinale*	Antioxidant (DPPH: IC_50_ 78.2 μM)	[65]
**115**	*p*-Hydroxyphenethyl *trans*-ferulate	Phenylpropanoids	*D. loddigesii*	Antioxidant	[49]
**116**	*n*-Tetracosyl *trans*-ferulate	Phenylpropanoids	*D. moniliforme*	Antioxidant	[54]
**117**	*n*-Pentacosyl *trans*-ferulate	Phenylpropanoids	*D. moniliforme*	Antioxidant	[54]
**118**	(−)-Pinoresinol	Lignans	*D. loddigesii*	Anti-inflammatory (NO: IC_50_ 89.5 μM)	[19]
**119**	Pinoresinol	Lignans	*D. nobile*	Antioxidant (DPPH: IC_50_ 60.6 μM)	[57]
			*Dendrobium* cv. Sonia	Anti-inflammatory (NO: IC_50_ 26.3 μM)	[29]
**120**	(−)-Medioresinol	Lignans	*D. loddigesii*	Anti-inflammatory (NO: IC_50_ 5.0 μM)	[19]
**121**	Medioresinol	Lignans	*D. nobile*	Antioxidant (DPPH: IC_50_ 27.9 μM)	[57]
**122**	Syringaresinol	Lignans	*D. loddigesii*	Anti-inflammatory (NO: IC_50_ 1.9 μM) and antioxidant (IC_50_ 31.1 μM)	[20]
			*D. nobile*	Antioxidant (DPPH: IC_50_ 9.8 μM)	[57]
			*D. secundum*	Antioxidant (DPPH: IC_50_ 11.4 μM)	[70]
			*Dendrobium* cv. Sonia	Anti-inflammatory (NO: IC_50_ 27.7 μM)	[29]
**123**	Lirioresinol A	Lignans	*D. nobile*	Antioxidant (DPPH: IC_50_ 30.9 μM)	[57]
**124**	Sesqui-illisimonan A	Lignans	*Dendrobium* cv. Sonia	Anti-inflammatory (NO: IC_50_ 31.6 μM)	[29]
**125**	Dendrocoumarin	Benzocoumarins	*D. nobile*	Antibacterial (*Staphylococcus aureus*: MIC 2.5 μg/mL; *Micrococcus tetragenus*: MIC 5.0 μg/mL)	[36]
**126**	Itolide A	Benzocoumarins	*D. nobile*	Antibacterial (*Staphylococcus aureus*: MIC 2.5 μg/mL; *Micrococcus tetragenus*: MIC 5.0 μg/mL)	[36]
**127**	Dendroflorin	Fluorenones	*D. nobile*	Anti-inflammatory (NO: IC_50_ 13.4 μg/mL) and antioxidant (DPPH: IC_50_ 16.2 μM)	[23]
			*D. palpebrae*	Antioxidant	[69]
**128**	Nobilone	Fluorenones	*D. nobile*	Anti-inflammatory (NO: IC_50_ 38.1 μg/mL)	[23]
**129**	3,6,9-Trihydroxy-3,4-dihydroanthracen-1-(2*H*)-one	Anthracenes	*D. loddigesii*	Anti-inflammatory (NO: IC_50_ 43.8 μM) and antioxidant (DPPH: IC_50_ 22.8 μM)	[20]
**130**	3-Hydroxy-4-methoxyphenylethanol	Phenylethanoids	*D. nobile*	Antioxidant (DPPH: IC_50_ 64.5 μM)	[56]
**131**	Syringic acid	Benzoic acid derivatives	*D. nobile*	Antioxidant (DPPH: IC_50_ 8.1 μM)	[56]
			*D. officinale*	Antioxidant (DPPH: IC_50_ 36.5 μM)	[65]

^a^Pharmacological data with EC_50_, IC_50,_ or MIC values are presented herein. Other data can be found in the text

### Anti-Inflammatory Activity

Skin inflammation is the most common complaint of those suffering from dermatological diseases. Inflammatory skin diseases are divided into acute and chronic conditions. Acute skin inflammation is associated with occasional rashes, itching and skin redness and may be caused by ultraviolet or ionizing radiation, allergens or chemical irritants. Chronic inflammatory skin diseases include atopic dermatitis (such as eczema), seborrheic dermatitis, psoriasis, and rosacea. Chronic inflammatory skin diseases may lead to significant and serious disruption of skin immunity [8].

#### *Dendrobium chrysanthum* Wall. ex Lindl.

A phenanthrene, dendrochrysanene (**75**), was isolated from the stems of *D. chrysanthum* collected from Yunnan, China. This compound significantly suppressed the mRNA levels of TNF-*α*, IL-8, IL10, and iNOS in murine peritoneal macrophages at a concentration of 11.2 μg/mL. The compound may be a potentially useful new anti-inflammatory agent [9].

#### *Dendrobium crepidatum* Lindl. & Paxton

A research was conducted on *D. crepidatum* stems collected from Yunnan, China. Total alkaloids (yield, 2.3%) were obtained from *D. crepidadum* stems, which exhibited inhibitory effects on nitric oxide (NO) production in lipopolysaccharide (LPS)-activated mouse peritoneal macrophages, with an IC_50_ value of 18.7 μg/mL. The active alkaloids were found to be dendrocrepidine A (**85**; IC_50_, 39.8 μM), dendrocrepidine B (**93**; IC_50_, 51.8 μM), dendrocrepidine C (**94**; IC_50_, 29.7 μM), dendrocrepidine D (**95**; IC_50_, 40.1 μM), dendrocrepidine E (**96**; IC_50_, 35.2 μM), (±)-dendrocrepidine F (IC_50_, 38.4 μM), (−)-dendrocrepidine F (**97**; IC_50_, 13.3 μM), and (+)-dendrocrepidine F (**98**; IC_50_, 42.7 μM) [10]. In another study, the racemic mixture of (±)-homocrepidine A was separated into a pair of enantiomers, (+)-homocrepidine A (**82**) and (−)-homocrepidine A (**83**). (±)-Homocrepidine A, (+)-homocrepidine A, (−)-homocrepidine A, and homocrepidine B (**84**) also inhibited NO production with IC_50_ values of 14.7, 3.6, 22.8, and 27.6 μM, respectively, as compared with the positive control indomethacin (IC_50_, 42.2 μM) [11]. In a very recent study, (±)-dendrocrepidine A (IC_50_, 63.8 μM), (+)-dendrocrepidine A (**85**; IC_50_, 16.1 μM), dendrocrepidine B (**86**; IC_50_, 14.3 μM), (±)-crepidine (IC_50_, 51.1 μM), (−)-crepidine (**87**; IC_50_, 29.9 μM), (+)-crepidine (**88**; IC_50_, 81.9 μM), (±)-dendrocrepidine A (IC_50_, 27.5 μM), (−)-dendrocrepidine A (**89**; IC_50_, 18.5 μM), (+)-dendrocrepidine A (**90**; IC_50_, 30.2 μM), (±)-isocrepidamine (IC_50_, 27.4 μM), (−)-isocrepidamine (**91**; IC_50_, 16.3 μM), and (+)-isocrepidamine (**92**; IC_50_, 73.0 μM) also exhibited inhibitory effects on NO production. Dexamethasone (IC_50_, 47.0 μM) was used as a positive control [12].

#### *Dendrobium denneanum* Kerr.

A series of phenanthrene derivatives were isolated from *D. denneanum* stems collected from Sichuan, China, and 2,5-dihydroxy-4-methoxy-phenanthrene 2-*O*-*β*-d-glucopyranoside (**76**; IC_50_, 4.6 μM), 2,5-dihydroxy-4-methoxy-phenanthrene 2-*O*-*β*-d-apiofuranosyl-(1 → 6)-*β*-d-glucopyranoside (**77**; IC_50_, 16.9 μM), 2,5-dihydroxy-4-methoxy-phenanthrene 2-*O*-*α*-l-rhamnopyranosyl-(1 → 6)-*β*-d-glucopyranoside (**78**; IC_50_, 41.5 μM), (*S*)-5-methoxy-2,4,7,9-tetrahydroxy-9,10-dihydrophenanthrene (**66**; IC_50_, 3.1 μM), (*S*)-4-methoxy-2,5,7,9-tetrahydroxy-9,10-dihydrophenanthrene (**67**; IC_50_, 4.2 μM), (9*R*)-1,2,5,9-tetrahydroxy-9,10-dihydrophenanthrene 5-*O*-*β*-d-glucopyranoside (**79**; IC_50_, 0.7 μM), 4-methoxyphenanthrene-2,5-diol (moscatin, **45**; IC_50_, 6.3 μM), (*S*)-4-methoxy-9,10-dihydrophenanthrene-2,5,9-triol (**65**; IC_50_, 27.4 μM), 2-methoxy-9,10-dihydrophenanthrene-4,5-diol (**57**; IC_50_, 7.6 μM), and 9,10-dihydrophenanthrene-2,4,7-triol (**56**; IC_50_, 32.7 μM) inhibited NO production in LPS-activated mouse macrophage RAW264.7 cells, as compared with the positive control curcumin (IC_50_, 6.2 μM) [13].

#### *Dendrobium findlayanum* C.S.P. Parish & Rchb.f.

Seco-dendrobine-type alkaloids and phenolics were isolated from *D. findlayanum* stems collected from Yunnan, China. Bibenzyl 6ʺ-de-*O*-methyldendrofindlaphenol A (**38**) inhibited NO production in RAW 264.7 cells with an IC_50_ value of 21.4 μM. MG-132 (IC_50_, 0.2 μM) was used as a positive control [14].

#### *Dendrobium heterocarpum* Lindl.

3-Hydroxy-4ʹ,5-dimethoxybibenzyl (**4**), gigantol (**7**), and dendrocandin I (**39**) were isolated from whole plants of *D. heterocarpum* collected from Yunnan, China, and these compounds reduced nitric oxide (NO) production in LPS-activated mouse macrophage RAW264.7 cells. At a concentration of 25 μM, the inhibition percentages of these three compounds were 51, 52, and 59%, respectively. NG-Monomethyl-L-arginine (L-NMMA) was used as a positive control (IC_50_, 34 μM) [15].

#### *Dendrobium huoshanense* Z.Z. Tang & S.J. Cheng

*Dendrobium huoshanense* Z.Z. Tang & S.J. Cheng was not accepted by “Plants of the World *online*.” However, because this name is used in the *Chinese Pharmacopoeia* (2020 edition) [16], it is cited in this review.

A pilot study was conducted to evaluate the clinical and immunomodulatory effects of an orally administered extract of *D. huoshanense* leaves and stems in children with moderate to severe recalcitrant atopic dermatitis (AD). AD is a common inflammatory skin disorder for which few safe and effective systemic treatments are available. Twenty-seven patients aged 4–18 years with AD who did not respond to topical therapy were treated with polysaccharides derived from *D. huoshanense* for 4 weeks and were followed-up for another 4 weeks. The results showed that the polysaccharide from *D. huoshanense* reduced the levels of some cytokines associated with AD and had beneficial effects on symptoms. No serious adverse effects occurred when the polysaccharide was administered orally for 4 weeks [17].

#### *Dendrobium loddigesii* Rolfe

Shihunine (**80**) was isolated from *D. loddigesii* stems collected from Yunnan, China. The alkaloid showed anti-inflammatory activity using the method of NO production the polysaccharide in RAW 264.7 cells activated by LPS with an IC_50_ value of 11.5 μg/mL. L-NG-monomethyl arginine citrate (IC_50_ 7.2 μg/mL) was used as a positive control [18].

Phenanthrenes and bibenzyls were isolated from *D. loddigesii* stems collected from Guangdong, China. These compounds were evaluated for their inhibitory activities against NO production. Loddigesiinol A (**52**; IC_50_, 2.6 μM), moscatin (plicatol B, **45**; IC_50_, 6.4 μM), 5-hydroxy-2,4-dimethoxyphenanthrene (**46**; IC_50_, 5.3 μM), lusianthridin (**54**; IC_50_, 4.6 μM), rotundatin (**64**, plicatol C; IC_50,_ 29.1 μM), hircinol (**55**; IC_50_, 29.2 μM), loddigesiinol B (**71**; IC_50_, 10.9 μM), loddigesiinol D (**26**; IC_50_, 69.7 μM), (−)-pinoresinol (**118**; IC_50_, 89.5 μM), and (−)-medioresinol (**120**; IC_50_, 5.0 μM) inhibited NO production, as compared with the positive controls aminoguanidine (IC_50_, 17.5 μM) and resveratrol (IC_50_, 22.0 μM) [19].

Fifteen compounds were isolated from a preparation of *D. loddigesii* originating in Yunnan, China. Chrysotoxol A (**74**; IC_50,_ 10.9 μM), neoechinulin A (**99**; IC_50_, 50.0 μM), 3,6,9-trihydroxy-3,4-dihydroanthracen-1-(2*H*)-one (**129**; IC_50_, 43.8 μM), 4,4ʹ-dihydroxy-3,5-dimethoxybibenzyl (**5**; IC_50_, 49.3 μM), naringenin (**104**; IC_50_, 26.9 μM), 5,4ʹ-dihydroxy-7,3ʹ,5ʹ-trimethoxyflavanone (**106**; IC_50_, 24.9 μM), 5,7,4ʹ-trihydroxy-3ʹ,5ʹ-dimethoxyflavanone (IC_50_, 19.1 μM), batatasin III (**1**; IC_50_, 21.9 μM), 3,3ʹ,5-trihydroxybibenzyl (**2**; IC_50_, 13.1 μM), trigonopol B (**28**; IC_50_, 26.3 μM), syringaresinol (**122**; IC_50_, 1.9 μM), 9,10-dihydrophenanthrene-2,4,7-triol (**56**; IC_50_, 8.6 μM), loddigesiinol B (**71**; IC_50_, 9.9 μM), loddigesiinol I (**72**; IC_50_, 7.5 μM), and loddigesiinol J (**73**; IC_50_, 14.6 μM) inhibited NO production in RAW 264.7 cells activated by LPS when compared with the positive control L-NMMA (IC_50_, 29.0 μM) [20].

#### *Dendrobium moniliforme* (L.) Sw.

Denbinobin (**68**) was isolated from *D. moniliforme* stems. At 1 μM, this compound significantly inhibited the formation of TNF-*α* and prostaglandin E_2_ (PGE_2_) (about 62 and 43% inhibition, respectively) in RAW264.7 cells stimulated with 1 μg/mL of LPS. In N9 cells (murine microglial cell line) stimulated with 10 ng/mL of LPS plus 10 unit/mL of interferon-*γ* (IFN-*γ*), denbinobin (3 μM) reduced TNF-*α* and nitrite formation (about 70 and 44% inhibition, respectively) [21].

#### *Dendrobium nobile* Lindl.

An alkaloid, anosmine (**81**), was isolated from *D. nobile*, which was purchased from Guangzhou, China. The compound exhibited anti-inflammatory activity using the method of NO production inhibition in RAW 264.7 cells activated by LPS with an IC_50_ value of 16.1 μg/mL, which was compared with L-NG-monomethyl arginine citrate (IC_50_, 7.2 μg/mL) as a positive control [18].

Phenanthrenes from the methanolic extract of *D. nobile* stems were evaluated for their potential to inhibit LPS-induced production of NO in murine macrophage RAW 264.7 cells. 3,4,8-Trimethoxyphenanthrene-2,5-diol (**51**; IC_50_, 20.4 μM), hircinol (**55**; IC_50_, 26.4 μM), erianthridin (**60**; IC_50_, 19.5 μM), ephemeranthol A (**63**; IC_50_, 12.0 μM), 5,7-dimethoxyphenanthrene-2,6-diol (**50**; IC_50_, 35.7 μM), moscatilin (**9**; IC_50_, 27.6 μM), coelonin (**58**; IC_50_, 10.2 μM), flavanthridin (**61**; IC_50_, 34.1 μM), epheneranthol C (**62**; IC_50_, 17.6 μM), lusianthridin (**54**; IC_50_, 9.6 μM), and fimbriol B (**48**; IC_50_, 28.9 μM) inhibited NO production. Aminoguanidine (IC_50_, 17.5 μM) was used as a positive control [22].

Nobilin D (**21**; IC_50,_ 15.3 μM), nobilin E (**33**; IC_50_, 19.2 μM), nobilone (**128**; IC_50_, 38.1 μM), chrysotobibenzyl (**16**; IC_50_, 48.2 μM), moscatilin (**9**; IC_50_, 36.8 μM), gigantol (**7**; IC_50_, 32.9 μM), and dendroflorin (**127**; IC_50_, 13.4 μM) from *D. nobile* stems collected from Yunnan, China, exhibited inhibitory effects on NO production in the murine macrophage-like cell line RAW264.7 activated by LPS and IFN-*γ*. Resveratrol (IC_50_, 23.5 μM) was used as a positive control [23].

Ephemeranthol A (**63**) was isolated from *D. nobile* stems, and its anti-inflammatory activity was evaluated in Raw 264.7 cells. This compound reduced NO production in a dose-dependent manner. Notably, with pretreatment with 12.5 μg/mL ephemeranthol A, NO production decreased to the level of the cell-only control [24].

#### *Dendrobium officinale* Kimura & Migo

Vicenin 2 (vicenin II, **108**) was suggested to be a common component in *D. officinale* leaves of different origins [25]. This di-*C*-glucosylflavone was synthesized, and it inhibited TNF-*α* expression and NO production with IC_50_ values of 6.8 and 3.9 μM, respectively, as compared with the positive control apigenin (IC_50_ = 18.5 and 19.0 μM, respectively) [26].

Two types of polysaccharides in *D. officinale* leaves, DLP-1 and DLP-2, were obtained by hot water extraction. DLP-1 (5 μg/mL) and DLP-2 (50 μg/mL) were shown to be effective in protecting THP-1 cells, a human leukemia monocytic cell line, from LPS-stimulated cytotoxicity and inhibited reactive oxygen species formation. In addition, both DLP-1 (5 μg/mL) and DLP-2 (50 μg/mL) significantly suppressed TLR4, myeloid differentiation factor (MyD88), and tumor necrosis factor receptor-associated factor-6 (TRAF-6) mRNA and protein expression in LPS-stimulated THP-1 cells [27].

#### *Dendrobium parishii* H. Low

(−)-Dendroparishiol (**36**) from the whole plant of *D. parishii* collected from Thailand was evaluated for its anti-inflammatory effects in LPS-stimulated RAW264.7 murine macrophage cells. At 12.5, 25 and 50 μg/mL, (−)-dendroparishiol reduced the expression of iNOS and COX-2 in LPS-treated RAW264.7 cells [28].

#### *Dendrobium* cv. Sonia

Ten compounds were isolated from *Dendrobium* cv. Sonia stems collected from Nanjing, China, and their anti-inflammatory activities were evaluated. 6-Feruloyloxyhexanoic ester (**112**; IC_50_, 29.6 μM), pinoresinol (**119**; IC_50_, 26.3 μM), syringaresinol (**122**; IC_50_, 27.7 μM), and sesqui-illisimonan A (**124**; IC_50_, 31.6 μM) exhibited inhibitory activities on NO production. Aminoguanidine (IC_50_, 27.3 μM) was used as a positive control [29].

#### *Dendrobium tosaense* Makino

Via oral administration at dosages of 30, 100, and 300 mg/kg for one week in an atopic dermatitis murine model, the standardized ethyl acetate extract of cultured *D. tosaense* stems protected mice from OVA/TNCB-induced skin lesions of atopic dermatitis [30].

### Antimicrobial Activity

Microorganisms can cause skin infections, such as carbuncles, furuncles, cellulitis, impetigo, boils (*Staphylococcus aureus*), folliculitis (*S. aureus*, *Pseudomonas aeruginosa*), ringworm (*Microsporum* spp., *Epidermophyton* spp., and *Trichophyton* spp.), acne (*Propionibacterium acnes*), and foot odor (*Brevibacterium* spp.) [31].

Gigantol (**7**) has been found in 33 *Dendrobium* species [32]. This compound showed inhibitory activity against *Staphylococcus aureus* with an MIC value of 82.2 μg/mL [33].

Sortase A (srtA), a transpeptidase in gram-positive bacteria, can anchor surface proteins that play a vital role in the pathogenesis of these bacteria. SrtA is known as a potential antivirulent drug target to treat bacterial infections. Erianin (**14**) was isolated from *D. chrysotoxum* stems [34]. This compound could inhibit the activity of srtA in vitro with an IC_50_ value of 20.9 μg/mL [35].

Dendrocoumarin (**125**) and itolide A (**126**) from *D. nobile* stems collected from Hainan, China, showed antibacterial activities against *Staphylococcus aureus* with the same MIC value of 2.5 μg/mL and *Micrococcus tetragenus* with the same MIC value of 5.0 μg/mL. The positive control was ciprofloxacin, with MIC values of 0.6 and 0.3 μg/mL for the two bacteria, respectively [36].

### Antioxidant and Antiaging Effects

The skin shows obvious signs of aging due to age, ultraviolet radiation exposure, and chemical pollution [37]. The changes in the skin are among the most visible signs of aging, including wrinkles, sagging skin, age spots dryness, and the loss of fat, which cause the skin to lose its natural smoothness [38]. The sum of the deleterious free radical reactions is a major contributor to the aging process [39]. In intrinsically aged skin, the quantity of dermal collagen decreases, and elastin accumulates structural abnormalities [40].

#### *Dendrobium amoenum* Wall. Ex Lindl.

Chloroform and acetone extracts of *D. amoenum* stems collected from Nepal showed 2,2-diphenyl-1-picrylhydrazyl (DPPH) free radical scavenging activities with IC_50_ values of 36.5 and 53.2 μg/mL, respectively [41].

#### *Dendrobium aphyllum* (Roxb.) C.E.C. Fisch.

Aphyllone B (**17**) from the stems of *D. aphyllum* collected from Yunnan, China, possessed DPPH radical scavenging activity with a scavenging percentage of 88% at a concentration of 100 μg/mL [42].

#### *Dendrobium crepidatum* Lindl. & Paxton

Ethanol and acetone extracts of *D. crepidatum* stems collected from Nepal showed DPPH free radical scavenging activities with IC_50_ values of 73.9 and 99.4 μg/mL, respectively, as compared with the positive control ascorbic acid (IC_50_, 38.2 μg/mL) [43].

#### *Dendrobium denneanum* Kerr

The ethanolic extract of *D. denneanum* [syn. *D. aurantiacum* var. *denneanum* (Kerr) Z.H. Tsi] stems collected from Yunnan, China, exhibited DPPH radical scavenging activity with an IC_50_ value of 92.6 μg/mL and was compared with *α*-tocopherol (IC_50_, 25 μg/mL) as a positive control. Three compounds were obtained by bioguided isolation. Unfortunately, the activities of these compounds were weaker than those of the crude extract [44].

A bibenzyl-rich fraction from *D. denneanum* stems collected from Sichuan, China, exhibited DPPH scavenging activity with an EC_50_ of 62.8 μg/mL. Vitamin C (EC_50_, 3.4 μg/mL) was used as a positive control [45].

#### *Dendrobium denudans* D. Don

The methanol extract of *D. denudans* stems collected from India showed in vitro antioxidant activity by a reducing power assay with an IC_50_ value of 10.1 μg/mL. Ascorbic acid (IC_50_, 3.9 μg/mL) was used as a positive control [46].

#### *Dendrobium draconis* Rchb.f.

5-Methoxy-7-hydroxy-9,10-dihydro-1,4-phenanthrenequinone (**69**; IC_50_, 283.3 μM or 72.6 μg/mL), hircinol (**55**; IC_50_, 22.3 μM), gigantol (**7**; IC_50_, 17.7 μM), and 7-methoxy-9,10-dihydrophenanthrene-2,4,5-triol (**59**; IC_50_, 10.2 μM) were isolated from *D. draconis* stems collected from Thailand and exhibited DPPH radical scavenging activities. Quercetin (IC_50_, 2.4 μM) and Trolox (IC_50_, 11.7 μM) were used as positive controls [47].

#### *Dendrobium huoshanense* Z.Z. Tang & S.J. Cheng

A polyphenol extract was obtained from *D. huoshanense* collected from Anhui, China, and the extract exhibited DPPH and 2,2ʹ-azino-bis(3-ethylbenzothiazoline-6-sulfonic acid) diammonium salt (ABTS) radical scavenging activities with IC_50_ values of 57 and 27 μg/mL, respectively, as compared with the positive control vitamin C [48].

#### *Dendrobium loddigesii* Rolfe

A series of phenolic compounds were found in *D. loddigesii* collected from Yunnan, China. From its stems, *threo*-7-*O*-ethyl-9-*O*-(4-hydroxyphenyl)propionyl-guaiacylglycerol (**113**), crepidatin (**15**), moscatilin (**9**), 4,5,4′-trihydroxy-3,3′-dimethoxybibenzyl (**13**), 4′,5-dihydroxy-3,3′-dimethoxybibenzyl (gigantol, **7**), tristin (**8**), dihydroconiferyl dihydro-*p*-coumarate (**114**), and *p*-hydroxyphenethyl *trans*-ferulate (**115**) were obtained, and these compounds exhibited significant activities, with DPPH scavenging capacities ranging from 89 to 94% at 100 μg/mL, as compared with the positive control Trolox, which led to an inhibition of 96% at a concentration of 25 μg/mL. Batatasin III (**1**) significantly stimulated the collagen production activity of human dermal fibroblasts-adult (HDFa) (EC_50_ 3.2 μg/mL) and was compared with TGF-*β* as a positive control, with an inhibition of 66% at a concentration of 0.01 μg/mL [49]. From complete plants, moscatin (**45**), 9,10-dihydrophenanthrene-2,4,7-triol (**56**), and crepidatuol B (**27**) were obtained, and these compounds showed significant activities, with DPPH scavenging capacities ranging from 84 to 95% at 100 μg/mL, as compared with the positive control Trolox, which led to an inhibition of 96% at a concentration of 25 μg/mL [50].

Phenanthrenes and bibenzyls were isolated from *D. loddigesii* stems collected from Guangdong, China. Loddigesiinol A (**52**; IC_50_, 26.1 μM), moscatin (**45**; IC_50_, 59.8 μM), lusianthridin (**54**; IC_50_, 62.2 μM), and loddigesiinol C (**25**; IC_50_, 23.7 μM) showed activities using the DPPH-scavenging assay, as compared to the positive controls resveratrol (IC_50_, 28.7 μM) and aminoguanidine (IC_50_, 21.7 μM) [19].

Fifteen compounds were isolated from a preparation of *D. loddigesii* stems collected from Yunnan, China. Chrysotoxol A (**74**; IC_50_, 23.2 μM), 3,6,9-trihydroxy-3,4-dihydroanthracen-1-(2*H*)-one (**129**; IC_50_, 22.8 μM), 4,4ʹ-dihydroxy-3,5-dimethoxybibenzyl (**5**; IC_50_, 94.5 μM), 5,4ʹ-dihydroxy-7,3ʹ,5ʹ-trimethoxyflavanone (**106**; IC_50_, 227.7 μM or 78.9 μg/mL), batatasin III (**1**; IC_50_, 204.9 μM or 50.1 μg/mL), 3,3ʹ,5-trihydroxybibenzyl (**2**; IC_50_, 85.8 μM), trigonopol B (**28**; IC_50_, 60.1 μM), syringaresinol (**122**; IC_50_, 31.1 μM), and 9,10-dihydrophenanthrene-2,4,7-triol (**56**; IC_50_, 14.1 μM) showed DPPH radical scavenging activities. Vitamin C (IC_50,_ 29.8 μM) was used as a positive control [20].

#### *Dendrobium longicornu* Lindl.

The acetonic extract of *D. longicornu* stems collected from Nepal showed DPPH radical scavenging activity with an IC_50_ value of less than 100 μg/mL and was compared with ascorbic acid (IC_50_, < 50 μg/mL) as a positive control [51].

#### *Dendrobium macrostachyum* Lindl.

Ethanolic extracts of *D. macrostachyum* stems and leaves collected from India exhibited DPPH radical scavenging activities with IC_50_ values of 10.2 and 31.5 μg/mL, respectively, as compared with the positive control ascorbic acid (IC_50_, 18.4 μg/mL); these extracts also had ABTS radical scavenging activities with IC_50_ values of 31.5 and 49.1 μg/mL, respectively, as compared with the positive control ascorbic acid (IC_50_, 34.9 μg/mL). The activities of the ethanol extracts were better than those of petroleum ether, methanol, or water extracts [52].

#### *Dendrobium moniliforme* (L.) Sw.

The DPPH radical scavenging activities of hexane, chloroform, acetone, and ethanol extracts of *D. moniliforme* stems collected from Nepal were measured, showing IC_50_ values of 52.7, 42.4, 49.6, and 58.8 μg/mL, respectively, as compared with the positive control ascorbic acid (IC_50_, 38.2 μg/mL) [53].

Based on bioguided fractionation and isolation, a mixture of *n*-pentacosyl *trans*-ferulate (**117**) and *n*-tetracosyl *trans*-ferulate (**116**) (1:4) was obtained from tissue culture-raised plants of *D. moniliforme*. At 100 μg/mL, the mixture of the alkyl ferulates exhibited DPPH radical scavenging activity (inhibition ˃ 50%) [54].

#### *Dendrobium nobile* Lindl.

Flavonoids have been detected in ethyl acetate, *n*-butanol, and aqueous extracts of *D. nobile* leaves collected from Guangdong, China. These three extracts showed DPPH free radical scavenging activities with IC_50_ values of 21, 11, and 13 μg/mL, respectively, with Trolox used as a reference compound (IC_50_, 7 μg/mL) [55].

Bibenzyls and other phenolic compounds were isolated from *D. nobile* stems collected from Yunnan, China. Nobilin A (**22**; IC_50_, 87.1 μM), nobilin B (**23**; IC_50_, 32.2 μM), nobilin C (**24**; IC_50_, 136.0 μM or 47.4 μg/mL), dihydroconiferyl alcohol (**109**; IC_50_, 50.9 μM), coniferylaldehyde (**110**; IC_50_, 127.9 μM or 22.8 μg/mL), 3-hydroxy-4-methoxyphenylethanol (**130**; IC_50_, 64.5 μM), and syringic acid (**131**; IC_50_, 8.1 μM) exhibited DPPH radical scavenging activities. Vitamin C (IC_50_, 18.0 μM) and BHT (IC_50_, 90.9 μM) were used as positive controls [56].

Nobilin D (**21**; IC_50_, 19.9 μM), nobilin E (**33**; IC_50_, 21.0 μM), crepidatin (**15**; IC_50_, 21.8 μM), dendrobin A (**10**; IC_50_, 40.3 μM), chrysotoxine (**11**; IC_50_, 14.0 μM), moscatilin (**9**; IC_50_,14.5 μM), gigantol (**7**; IC_50_ 56.4 μM), and dendroflorin (**127**; IC_50_, 16.2 μM) from *D. nobile* stems exhibited DPPH radical scavenging activities. Vitamin C (IC_50_, 18.0 μM) was used as a positive control [23].

Phenanthrenes and lignans were isolated from *D. nobile* stems in Yunnan, China. Fimbriatone (**70**; IC_50_, 144.5 μM or 40.8 μg/mL), confusarin (**47**; IC_50_, 12.9 μM), flavanthrinin (**49**; IIC_50_, 35.7 μM), 2,5-dihydroxy-4,9-dimethoxyphenanthrene (**53**; IC_50_, 34.8 μM), 5,7-dimethoxyphenanthrene-2,6-diol (**50**; IC_50_, 29.7 μM), syringaresinol (**122**; IC_50_, 9.8 μM), pinoresinol (**119**; IC_50_, 60.6 μM), medioresinol (**121**; IC_50_, 27.9 μM), and lirioresinol A (**123**; IC_50_, 30.9 μM) exhibited DPPH radical scavenging activities. Vitamin C (IC_50_, 18.0 μM) and BHT (IC_50_, 90.9 μM) were used as positive controls. For all phenanthrenes and lignans, an electron-donating methoxy group in the *ortho* position that donates to the phenolic hydroxy group exhibits enhanced antioxidant activity [57].

#### *Dendrobium officinale* Kimura & Migo

Ethanolic extracts of *D. officinale* flowers, leaves, and stems collected from Zhejiang, China, in which the total flavonoid contents measured were 1.8, 0.25, and 0.052%, respectively, were prepared. These extracts showed DPPH scavenging activities with IC_50_ values of 0.2 μg/mL, 17.4 μg/mL, and 10.4 μg/mL, respectively, as compared with the positive control vitamin C (IC_50_, 7.5 μg/mL) [58].

The chloroform extract of *D. officinale* stems collected from Yunnan, China, exhibited ABTS radical scavenging activity with an IC_50_ value of 88.8 μg/mL [59].

The antioxidant activities of *D. officinale* (Syn. *D. candidum*) collected from different areas of Hainan, China, were compared. Ethanolic extracts of plant samples collected from Sanya, Qiongzhong, and Baoting exhibited the best DPPH radical scavenging activities, with IC_50_ values of 15.9, 20.2, and 78.7 μg/mL, respectively [60].

A series of bibenzyls were isolated from *D. officinale* (syn. *D. candidum*) stems collected from Zhejiang, China. Dendrocandin C (**18**; IC_50_, 34.2 μM), dendrocandin D (**19**; IC_50_, 34.5 μM), dendrocandin E (**12**; IC_50_, 15.6 μM) [61], dendrocandin F (**29**; IC_50_, 55.8 μM), dendrocandin G (**30**; IC_50_, 32.4 μM), dendrocandin H (**34**; IC_50_, 19.8 μM), dendrocandin I (**39**; IC_50_, 21.3 μM) [62], dendrocandin J (**31**; IC_50_, 36.8 μM), dendrocandin K (**32**; IC_50_, 70.2 μM), dendrocandin L (**35**; IC_50_, 45.0 μM), dendrocandin M (**37**; IC_50_, 60.5 μM), dendrocandin N (**42**; IC_50_, 87.6 μM), dendrocandin O (**43**; IC_50_, 50.4 μM), dendrocandin P (**40**; IC_50_, 22.3 μM), dendrocandin Q (**41**; IC_50_, 30.3 μM) [63], and (*S*)-3,4,*α*-trihydroxy-5,4ʹ-dimethoxybibenzyl (**20**; IC_50_,IC_50_, 32.3 μM) exhibited DPPH radical scavenging activities [64]. Vitamin C (IC_50_, 23.2 μM) was used as a positive control [61–64].

Moupinamide (**100**; IC_50_, 53.8 μM), dihydroconiferyl dihydro-*p*-cumarate (**114**; IC_50_, 78.2 μM), dihydroferuloyltyramine (**101**; IC_50_,113.5 μM or 35.8 μg/mL), syringic acid (**131**; IC_50_, 36.5 μM), ferulic acid (**111**; IC_50_, 64.9 μM), and 3′,5,5′,7-tetrahydroxyflavanone (**105**; IC_50_, 29.8 μM) from *D. officinale* (syn. *D. catenatum*) stems collected from Zhejiang, China, showed DPPH free radical scavenging activities. Vitamin C (IC_50_, 23.2 μM) was used as a positive control [65].

Eight bibenzyls were isolated from *D. officinale* (syn. *D. catenatum*) stems collected from Shenzhen, China. Dendrocandin U (**44**), 3,4ʹ-dihydroxy-5-methoxybibenzyl (**3**), and 3,4ʹ,5-trihydroxy-3ʹ-methoxybibenzyl (tristin, **8**) exhibited significant ABTS radical scavenging activities with IC_50_ values of 10.0, 5.3, and 9.0 μM, respectively, as compared with the positive control vitamin C (IC_50_, 6.5 μM). 3,4ʹ,5-Trihydroxy-3ʹ-methoxybibenzyl also exhibited DPPH scavenging activity with an IC_50_ value of 34.5 μM. Vitamin C (IC_50_, 14.9 μM) was used as a positive control [66].

*D. officinale* protocorm powder in deionized water (10, 25 and 50 mg/mL, external administration) significantly reduced erythema and protected the skin from dryness in a hairless mouse model with UV irradiation-induced skin damage using matrixyl (10 mg/mL) as a positive control. This study demonstrated that *D. officinale* protocorms can inhibit photodamage in the skin of hairless mice [67].

An in vivo experiment using photoaged model mice was conducted. The results showed that the ultrafine powder and fine powder of *D. officinale* (syn. *D. candidum*) stems possess a certain preventive effect on photoaging, and the effect of ultrafine powder is better than that of fine powder [68].

#### *Dendrobium palpebrae* Lindl.

A fluorenone, dendroflorin (**127**), was isolated from *D. palpebrae* whole plants collected from Thailand. This compound significantly decreased ROS in H_2_O_2_-stimulated RAW264.7 cells in a dose-dependent manner (12.5–50 μg/mL) [69].

#### *Dendrobium secundum* (Blume) Lindl. ex Wall.

Five compounds were isolated from *D. secundum* stems collected from Thailand. 4,5,4ʹ-Trihydroxy-3,3ʹ-dimethoxybibenzyl (**13**; IC_50_ 15.9 μM), moscatilin (**9**; IC_50_ 5.1 μM), syringaresinol (**122**; IC_50_ 11.4 μM), and ferulic acid (**111**; IC_50_ 37.5 μM) exhibited DPPH free radical scavenging activities, which were compared with the positive controls quercetin and Trolox (IC_50_, 2.5 and 11.7 μM, respectively) [70].

#### *Dendrobium signatum* Rchb.f.

*D. signatum* leaves collected from Thailand were extracted with ethanol by sonication-maceration for 30 min. The extract showed DPPH radical scavenging activity, with a measured IC_50_ value of 97.2 μg/mL, with ascorbic acid used as a reference compound (IC_50_, 21.7 μg/mL) [71].

#### *Dendrobium speciosum* Sm.

Methanolic extract of *D. speciosum* leaves collected from Australia containing polyphenols (1.2%) and flavonoids (0.2%) showed DPPH scavenging activity, with a measured IC_50_ value of 26 μg/mL. Trolox was used as a reference compound (IC_50_, 20 μg/mL) [72].

#### *Dendrobium tosaense* Makino

The effects of methanolic extracts obtained from three *Dendrobium species* propagated in vitro on DPPH scavenging were investigated. The *D. tosaense* extract was the most active extract, with an IC_50_ value of 79.9 μg/mL, as compared with the positive control *α*-tocopherol (IC_50_, 58.2 μM) [73].

Based on bioguided fractionation and isolation, quercetin (**103**) was obtained from tissue culture-raised plants of *D. tosaense*. At 100 μg/mL, quercetin exhibited DPPH radical scavenging activity (inhibition ˃ 50%) [54].

#### *Dendrobium williamsonii* Day & Rchb.f.

Six compounds were isolated from *D. williamsonii* whole plants collected from Thailand and evaluated these isolates for their DPPH radical scavenging activities. 3,3ʹ-Dihydroxy-4,5-dimethoxybibenzyl (**6**), moscatilin (**9**), and apigenin (**102**) were active, with IC_50_ values of 19.5, 8.5, and 19.3 μM, respectively, as compared with the positive controls, quercetin (IC_50_, 8.3 μM) and vitamin C (IC_50_ 42.4 μM) [74].

### Anti-Psoriasis Activity

Psoriasis is a recurrent skin disease described as keratinocyte hyperproliferation and aberrant differentiation. At concentrations ranging from 12.5 nM to 50 nM, erianin (**14**) inhibited proliferation and induced apoptosis in a human keratinocyte cell line (HaCaT). Erianin could be recognized as a potential anti-psoriasis drug [75]. This compound was previously isolated from *D. chrysotoxum* [34].

### Hair Growth Promoting Effects

Alopecia is a skin disease characterized by reduced hair [76]. The condition has a strong influence on the mental and psychological health of patients [77].

In an in vivo experiment, C57BL/6 J mice were externally administered *D. officinale* (Guangdong, China) polysaccharides (DOP, 5.0 g/L) for 21 days. The average hair growth score and average quality of C57BL/6 J mice in the DOP group were significantly better than those in the control groups [78].

### Skin-Moisturizing Effects

When the water content in the stratum corneum drops to less than 10%, the skin appears dry, loses elasticity, and wrinkles and skin aging accelerates [77].

The moisture retention rate of *D. huoshanense* (Anhui, China) polysaccharide on human skin is significantly higher than that of glycerol after external administration for 6 h, 8 h and 12 h. The stimulus value of normal skin and damaged skin of rabbits is less than 0.5, indicating that *D. huoshanense* polysaccharides do not cause skin irritation [79].

Polysaccharides from white orchids (*Dendrobium* cv. Khao Sanan) flowers cultivated in Thailand were evaluated in vivo for their skin hydration efficacy in human volunteers. The efficacy of white orchid polysaccharides at 0.3% was noted to be superior in terms of skin hydration efficacy than sea weed polysaccharides at 0.2% [80].

An in vivo experiment was conducted to evaluate the moisturizing effects of a *D. nobile* stem extract on human skin. The moisturizing abilities after 30 min and 2 h were greater than 1 for the *D. nobile* stem extract (2.0%) [81].

An in vivo experiment showed that 20 μg/mL ethanolic extract of *D. officinale* collected from Yunnan, China, exhibited a skin moisturizing effect. After 2.5 h of use, skin hydrature increased by 16% compared with that before use (*P* < 0.05) [82].

### Tyrosinase-Inhibitory Activity

Tyrosinase inhibitors are used for hyperpigmentation and developing skin whitening agents.

3,3ʹ,5-Trihydroxybibenzyl (**2**) from *D. loddigesii* stems collected from Yunnan, China, revealed significant inhibitory activity against tyrosinase with an IC_50_ value of 37.9 μg/mL, as compared with the positive control kojic acid (IC_50_, 8.0 μg/mL) [49].

At concentrations of 12.5, 25 and 50 μg/mL, the CH_2_Cl_2_ extract of *D. moniliforme* leaves collected from South Korea inhibited melanogenesis in murine melanoma cells (B16F10), implying that *D. moniliforme* is effective against hyperpigmentation disorders and that it is considered a possible antimelanogenic agent in topical application [83].

Ethanolic extracts of *Dendrobium* cv. Sonia flowers and *Dendrobium* cv. Sonia pink flowers collected from Thailand showed tyrosinase inhibitory activities using l-tyrosine as a substrate. The IC_50_ values were 57.4 μg/mL and 83.2 μg/mL, respectively, as compared with the positive control kojic acid (IC_50_,151.7 μg/mL) [84].

## Conclusion

There are 22 *Dendrobium* species with traditional uses for treating dermatological disorders by local people in eight countries, and there are 131 compounds from *Dendrobium* plants reported to possess anti-inflammatory, antimicrobial, antioxidant, antiaging, anti-psoriasis, and tyrosinase-inhibitory activities, which implies that *Dendrobium* plants are important resources for the discovery of active compounds and the development of new drugs and cosmetics. However, only *D. crepidatum*, *D. denneanum*, *D. loddigesii*, *D. nobile*, and *D. officinale* have been extensively studied. More research on other *Dendrobium* species is needed.

The major active compounds found in *Dendrobium* species are phenanthrenes, alkaloids, flavonoids, phenylpropanoids, and lignans. Several compounds, such as loddigesiinol A, (*S*)-5-methoxy-2,4,7,9-tetrahydroxy-9,10-dihydrophenanthrene, (*S*)-4-methoxy-2,5,7,9-tetrahydroxy-9,10-dihydrophenanthrene, 2,5-dihydroxy-4-methoxy-phenanthrene 2-*O*-*β*-d-glucopyranoside, (9*R*)-1,2,5,9-tetrahydroxy-9,10-dihydrophenanthrene 5-*O*-*β*-d-glucopyranoside, (+)-homocrepidine A, and vicenin 2, have significant anti-inflammatory activities and inhibit NO production with IC_50_ values less than 5 μM, and these compounds are worthy of further study.
